# What Activities in Forests Are Beneficial for Human Health? A Systematic Review

**DOI:** 10.3390/ijerph19052692

**Published:** 2022-02-25

**Authors:** Sujin Park, Eunsoo Kim, Geonwoo Kim, Soojin Kim, Yeji Choi, Domyung Paek

**Affiliations:** 1 Forest Human Service Division, Future Forest Strategy Department, National Institute of Forest Science, Seoul 02455, Korea; snowshoe@korea.kr (S.P.); euncarp2@gmail.com (E.K.); bkim5020@korea.kr (G.K.); kimsoojinsj@korea.kr (S.K.); usmile.choi@gmail.com (Y.C.); 2 Department of Environmental Health Sciences, Graduate School of Public Health, Seoul National University, Seoul 08826, Korea; 3 Institute of Health and Environment, Seoul National University, Seoul 08826, Korea

**Keywords:** nature-based intervention, forest-based intervention, forest therapy, psychological outcome, physiological outcome, health promotion

## Abstract

Over the past decade, clinical trials of forest-based interventions have increased, leading to their recognition as preventive medicine. However, little is known about the differences in health effects according to the activity characteristics of interventions. This study aimed to understand the types of activities and their associated health effects to identify differences in health effects between activities. PubMed, PsycINFO, Web of Science, and Scopus databases were searched, and methodological quality was assessed using Cochrane ROB2. A total of 32 randomized controlled trials (RCTs) met the eligibility criteria. Health outcomes were collected from 6264 participants aged 6–98 years, and the sample size was 12–585. The Interventions were walking (n = 21), staying (n = 7), exercise (n = 4), indirect exposure (n = 4), and the activity time was between 10 and 240 min. Overall, walking showed consistent positive health effects, and there were differences in effects on anxiety and depression, cognitive function, stress hormone, and inflammation according to the activity. However, most of the included studies had a high risk of bias, and interventions were limited to specific activities, durations, and frequencies. Although a few limitations remain, the findings in this study are of great significance in providing the basis for the design of forest-based interventions.

## 1. Introduction

Health benefits of the natural environment have long been discussed, and in recent years there has been an increasing trend to link forestry and public health. Since the late 2000s, several countries in East Asia, Europe, and North America have started to utilize urban forests, peri-urban forests, and conservation areas to promote public health and well-being [1,2,3,4,5,6]. East Asian countries, such as Korea and Japan, have actively operated forest therapy programs under the name of “forest healing” or “forest bathing”. In the 1980s, forest bathing—taking in the atmosphere of the forest—emerged as a therapeutic method firstly in Japan. Since then, the physiological effects of forest environments have been investigated [7,8]. Similarly, the Korean government enacted the “Forestry Culture and Recreational Act and Forest Welfare Promotion Act”, defining “forest healing” as immune-strengthening and health-promoting activities utilizing various elements of the forest [9]. Moreover, Korea implemented the national qualification system for a forest healing instructor to train experts who develop and provide forest healing programs in the field [9]. In Europe, Finland has launched “MEIJÄN POLKU”, a large-scale transversal program supporting forest-based health promotion activities from 2017 to 2047 [10]. In North America, Canada, and the United States, the ‘Healthy Parks Healthy People’ program has recently been operated to improve public health by using various natural environments, including forests [11]. These national attempts can be referred to as “nature-based interventions” (NBIs), or highly specific forest-based interventions, in that they are designed activities or programs to enhance an individual’s experience in natural environments (i.e., forests or urban forests) to achieve specific health and well-being goals [12]. Forest-based interventions differ from mere experiences in forest settings, since they are designed activities or programs by experts to achieve direct health benefits [13].

Along with international efforts to utilize forests as preventive medicine, clinical trials on forest-based interventions have sharply increased. With respect to PubMed searches, research on health effects of forest-based interventions has been increasing since the 1990s, with 830 studies per year in the 2000s and greater than 3000 studies per year in recent times. The effects of forest-based interventions have been reported in various health domains such as cardiovascular function [14,15,16,17,18,19], immune system [16,17,20,21,22,23,24], endocrine system [25,26], and mental health [27,28,29,30]. Accordingly, there is also an increasing number of systematic reviews to summarize recent evidence and discover existing knowledge gaps.

Previous reviews have often focused on the overall health benefits of spending time in the forest [31], or the clinical outcomes of forest therapy programs [32,33]. Others have focused on one specific health effect, such as blood pressure [34,35], diabetes [35], stress recovery [36], and depression [37]. The current line of reviews have focused on investigating the health effects of forest-based interventions themselves rather than the effects of the composition of the interventions. Reviews have been conducted of certain forest-based programs such as immersive nature experiences [38] and green exercise [39]. However, a review of certain type of interventions provides fragmentary evidence; information about the health effect by activity characteristics are still limited. As a higher amount of clinical evidence accumulates, a higher nuanced review of forest-based interventions is required. It is necessary to identify common components of forest-based interventions and summarize how well the health impacts of each component are supported by clinical evidence.

Therefore, this review focused on the relationship between activities performed during forest-based intervention and health effects. To this end, this review collected empirical studies on forest-based interventions, divided the studies according to their activity components, and evaluated the comprehensive health effects of each type of activity. The purpose of this systematic review was to answer three questions: (1) what kind of activities were performed during the forest-based intervention in recent empirical studies, (2) what kind of health effects were associated with each activity component, and (3) how strong is the evidence for associations between the health effects and each activity components. By answering these questions, it is expected that this study will contribute to the development of strategies for designing forest-based interventions.

## 2. Materials and Methods

The current review was written according to the Preferred Reporting Items for Systematic Reviews and Meta-Analysis checklist [40,41]. The PRISMA statement for the current review can be found in Table S1. To provide credible evidence, the guidelines of the Cochrane Handbook for Systematic Reviews of Interventions version 6.2 were followed [42,43]. The study was registered in OSF database under the number DOI: 10.17605/OSF.IO/8NYVH. The authors attempted to gather all the relevant studies and reported the search strategy for obtaining reproducible search results. Moreover, studies that met this review’s eligibility criteria were included and their results were synthesized. The quality of each study was evaluated using the Cochrane risk of bias tool version 2 (RoB2).

### 2.1. PICOS and Eligibility Criteria

The research question was set by specifying the Population, Intervention, Comparison, Outcome, and Study design (PICOS). To classify the related studies, the study question and eligibility criteria based on the PICOS framework were established (Table 1).

### 2.2. Search Strategy

To develop keywords for searching relevant studies, previous systematic reviews on greenspace-based interventions that could be considered forest-based interventions [31,33,35,36,38,39] were referred to. Furthermore, the scope of the study design was limited to RCTs and randomized cross-over studies, as sufficient clinical controlled trials were found through pilot searches. Finally, search keywords to identify interventions, participants, outcomes, and study designs were selected (Table 2). The authors sought to identify all relevant studies that include any appropriate comparator to derive the health effects of forest-based interventions. Therefore, this review did not used keywords for comparators. Instead, keywords identifying study designs were used. Four databases were searched: PubMed, PsycINFO, Web of Science, and Scopus. All studies were published in English from January 2000 to February 2021.

### 2.3. Study Selection

**Figure 1:**
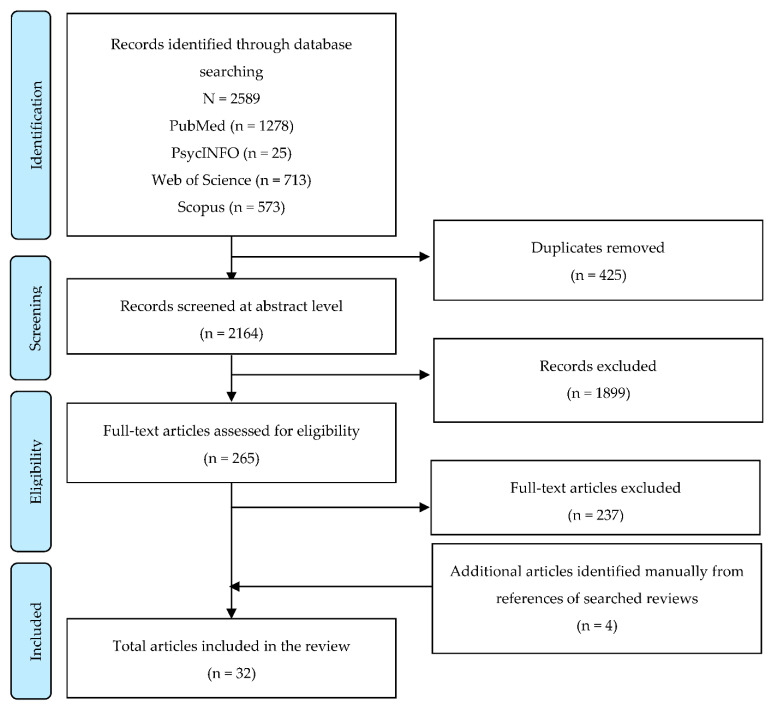
Flow diagram illustrating the selection process.

From the database search, a total of 2589 studies were found, with 1278 on PubMed, 25 on PsycINFO, 713 on Web of Science, and 573 on Scopus. The results were exported to EndNote Citation Manager software (version Endnote X9.3.3, Clarivate, Boston, MA, USA). After removing 425 duplicates, the titles and abstracts of the 2164 publications were reviewed. Two reviewers independently screened (E.K. and S.P.) the full text for 265 documents based on the eligibility criteria after removing 1899 nonrandomized studies or explicitly unrelated documents. The discrepancy during the screening process was resolved by two other investigators (S.K. and G.K.). By reviewing the references of the searched systematic reviews, four additional studies that met the eligibility criteria were identified and included. Overall, 32 studies were included in the review (Figure 1).

### 2.4. Data Extraction

Data were extracted from the documents by a single investigator (G.K. and E.K.) using the same data extraction form. The extracted data included study information (e.g., year of publication and author), samples (e.g., sample size, gender, participant characteristics, and age), intervention design (e.g., undertaken area, activity duration, and frequency), study design (study design and comparator), and outcome measurements.

### 2.5. Narrative Synthesis

In the current review, a narrative synthesis of the empirical evidence was conducted. Inspired by the reporting method of Mygind et al. [38]—one of the previous systematic reviews—the consistency of significant results of studies was assessed according to the activities conducted. The 32 included studies were divided into homogeneous groups according to the activity performed in the intervention, and the ratio of significant outcomes in each group was calculated. The results of the included studies were classified as having a significant positive effect on the health outcome (+), a mixed effect including both significant and nonsignificant effects on the positive health outcome (+/), a nonsignificant effect on the positive health outcome (/), or any negative health outcome (−). The percentage of the significant positive effect(%p) and of both the positive and mixed effects (%p + m) was calculated. Subsequently, the results of each group based on the different health domains were compared.

### 2.6. Methodological Quality

Along with synthesizing the empirical evidence, the quality of the included studies was investigated by assessing the risk of bias in the individual studies using the Cochrane RoB 2 tool in accordance with the latest version of the Cochrane Handbook for Systematic Reviews of Interventions version 6.2 [43]. The RoB 2 tool was adopted because it is the most comprehensive tool that can evaluate the risk of bias in both RCT and randomized cross-over studies [42]. Furthermore, the RoB 2 can evaluate all forms of bias that may occur in randomization, experimental design, study conduction process, and reporting. There are five areas of bias covered in the RoB 2 tool, which assesses bias that occurred during randomization (D1) by dropout (D2), due to missing data (D3), during measurement (D4), and by selecting results (D5). The overall risk of bias has the highest risk across the five domains. The evaluation in each domain was proposed as being “low risk”, “some concerns”, and “high risk” by algorithms with answers to signaling questions.

## 3. Results

### 3.1. Study Characteristics

A total of 6264 participants were reported in the 32 included studies, with the number of samples ranging from 12 to 585. In total, 21 studies reported results with small samples (≤60), and 11 studies reported results including samples ranging from 67 to 585. The age of participants ranged from 6 to 98 years, with 17 children in one study, 1048 young adults in 11 studies, 164 middle-aged adults in two studies, and 232 older adults in seven studies. In 11 studies, 744 adults participated in activities without age restrictions.

Overall, 10 of the included studies were conducted in urban forests, such as urban green spaces, city parks, and forests within walking distance. In total, 18 studies were conducted in remote forest areas, such as recreational forests, forest reserves, and wild forests. Moreover, four studies were conducted mainly indoors by indirect exposure using natural or audiovisual materials. The included studies reported quantified psychological (28 studies) or physiological (25 studies) health outcomes. Psychological outcomes included mood (n = 16), affect (n = 8), anxiety (n = 7), depression (n = 4), cognitive function (n = 8), well-being, and quality of life (n = 6). Physiological outcomes included nervous system (n = 19), stress hormone (n = 12), blood pressure (n = 12), cardiovascular disease (n = 6), inflammation (n = 7), oxidative stress and antioxidant (n = 9), immune function (n = 4), and pulmonary function (n = 1).

The activities conducted in the interventions were categorized into four activities: staying, walking, exercise, and indirect exposure. Staying (n = 7) referred to static activities in the forest, including sitting, viewing, watching, and relaxation sessions. Both walking and exercise are physical activities, but with different intensities. In addition, walking focuses on providing an experience by moving along a designated course, while exercise focuses on promoting physical activity. Walking (n = 21) referred to walking and exploring given places such as unhurried pace walks, leisurely walking, walking along a given course, instructed walking, and walking and observing surroundings. Exercise (n = 4) referred to activities composed of physical activity with a higher intensity, such as hiking and workout sessions. Indirect exposure (n = 4) referred to activities in which interventions were performed mainly indoors, utilizing audiovisual and natural materials. Two studies conducted both staying and walking in their intervention and reported the effects separately. One study reported a single result of walking, staying, and exercising. The duration of the interventions ranged from 10 min to 240 min. The main characteristics of the included studies are summarized in Table 3.

The distribution of psychological and physiological results showed differences according to activity. To examine how well evidence supported the health effects of each activity, the consistency by using ratio of significant outcomes was calculated (Table 4). Regarding psychological outcomes, the consistency of evidence was high in order of walking (%p = 66.7; %p + m = 100.0), staying (%p = 35.7; %p + m = 57.1), indirect exposure (%p = 23.1; %p + m = 38.5), exercise (%p = 12.5; %p + m = 25.0). Regarding physiological outcomes, the consistency of evidence was high in order of staying (%p = 50.0; %p + m = 66.7), walking (%p = 58.3; %p + m = 68.1), indirect exposure (%p = 50.0; %p + m = 50.0), exercise (%p = 0.0; %p + m = 50.0).

### 3.2. Psychological Outcomes

#### 3.2.1. Mood

Four studies assessed the effect of staying on subjective feeling [44], stress response symptoms [45], and burnout [46,47]. Tsunetsugu et al. [44] demonstrated that sitting in forests for 15 min and looking at landscapes significantly improved subjective feelings, including comfort, calm, and feeling refreshed compared with the urban environment. Im et al. [45] conducted staying activities in cities or forests for 2 h and found that staying in the forest partially mitigated psychological stress response symptom. Stigsdotter et al. [47] assessed 10 weeks of natural-based therapy in comparison with cognitive behavioral therapy. Both interventions showed a significant reduction in burnout (SMBQ) scores, indicating that natural-based therapy could be as effective as conventional treatments, while Sonntag-Öström et al. [46] reported no significant difference in burnout (SMBQ), stress, and fatigue between the waitlist control and intervention group, where participants regularly spent time alone in the forest for 11 weeks.

Ten studies examined the effect of walking on mood states [17,19,48,49,50,51], distress [52], perceived stress levels [53], and subjective feelings [44]. Song et al. [49,51] demonstrated that a 15 min walk in a well-managed artificial forest led to a significant decrease in negative mood—depression, anxiety, anger, fatigue, confusion—and a significant increase in vigor compared with walking in an urban environment. Similarly, Song et al. [50] reported significant reductions in negative mood—depression, anxiety, anger, fatigue, and confusion—and significant increases in vigor after a 17 min walk in forest compared with city walk. While three other studies reported partial improvements in mood states through forest walks. Mao et al. [17] identified significant improvements in depression, anxiety, anger, and confusion, but did not observe significant changes in fatigue and vigor. Mao et al. [48] identified significant improvements in depression, anxiety, anger, fatigue, and vigor, but did not confirm significant results in confusion. Mao et al. [19] identified significant improvements in depression, anger, confusion, and fatigue, but no significant changes were observed in anxiety and vigor. Ameli et al. [52] found that a 20 min walk in the forest garden significantly lowered distress level and improved mindful awareness compared with walking on an urban road. Koselka et al. [53] assigned the same subjects to walk across a forest and an urban roadside for 50 min on different dates, and observed the most consistent stress reduction in forest walking. Tsunetsugu et al. [44] found that walking in a forest for 15 min had a positive effect on subjective feelings—comfort, calm, and feeling refreshed—rather than walking in an urban environment.

Two studies investigated the effects of exercise on distress [54], tranquility, and fatigue [55]. One study compared the changes in distress levels of a six-month park prescription group with an indoor standard physical activity group; no significant difference was found [54]. Another study assessed the effect of 45 min of exercise for three days in the park and indoors; no significant difference in the improvement of tranquility and fatigue was found [55].

#### 3.2.2. Affect

One study assessed the effect of staying on affective states [56]. Rodiek [56] reported that staying in an outdoor garden for 2.5 h increased positive affect and reduced negative affect.

Three studies investigated the effect of walking on affective states [53,57,58]. Olafsdottir et al. [57] compared the affective states (PANAS) of a treadmill walking group with a forest walking group and demonstrated that forest walking significantly promoted positive affect and significantly improved negative affect. Koselka et al. [53] reported that a 50 min walk in the forest had a significant effect on reducing negative affect, but did not have a significant effect on positive affect compared with an urban road walk. Grazuleviciene et al. [58] found that walking in urban parks for 30 min for seven days was especially effective in reducing negative affect than walking on urban streets, but reported no significant change in positive affect.

One study assessed the effect of exercise on affective states. Calogiuri et al. [55] conducted a 45 min exercise over three days indoors or at parks. They found a significant improvement in positive affect was observed in the park exercise group, however, there was no significant difference in the improvement of negative affect.

Three studies examined the effects of indirect exposure to forests on affective states [59,60,61]. McAllister et al. [59] assigned subjects to forest video viewing groups, urban forest video viewing groups, and controls. They found that watching forest videos significantly improved positive and negative affect, whereas watching urban forest landscapes, only increased positive affect. Valtchanov et al. [60] conducted forest or abstract observation activities through virtual reality (VR) for 10 min and confirmed the significant effect of forest observation through VR on positive emotions, but did not confirm a significant effect on negative emotions. Golding et al. [61] conducted an intervention including viewing a slide show and reflecting for 11 min, and showed that watching forest scenery did not show any significant effect on positive affect, and even aroused negative effects.

#### 3.2.3. Anxiety and Depression

Two studies investigated the effects of staying on improving anxiety and depression. In one of the studies, observations were conducted indoors or in horticultural gardens for 2.5 h, and the variation in the Spielberger State-Trait Anxiety Inventory (STAI) score—a measure of anxiety state—was compared. It was reported that the effect of observing in the horticultural garden was not significant but tended to relieve anxiety [56]. Another study compared the results of the Hospital Anxiety and Depression Scale measurements—a measure of anxiety and depression—in groups of waitlist control with groups that regularly spent time in solitude in the forest for 11 weeks. The report showed no significant differences in depression and tension between the two groups [46].

Four studies examined the effects of walking on anxiety [49,53,62,63]. Song et al. [49] compared changes in the score of STAI after a 15 min walk in a city or an artificial forest and demonstrated that forest walks significantly lowered anxiety levels compared with city walks. Hassan et al. [62] reported that a 15 min walk in a forest was effective in mitigating anxiety, as the STAI score was significantly lower than the same length walks in the city. Koselka et al. [53] allowed the same participants to walk across a forest and urban roadside for 50 min on different dates and found that walking in the forest improved anxiety symptoms. Chun et al. [63] performed walking in a four-day program in forests and cities and compared the STAI scores. They found a significant improvement in all indicators by walking in a forest, especially the anxiety state reduction effect.

One study investigated the effect of indirect exposure to the forest on anxiety and depression [64]. Ng et al. [64] conducted a gardening session for three months using natural materials, and found no significant effect on the Self-rating Anxiety Scale and Self-rating Depression Scale scores compared with the waitlist control group.

#### 3.2.4. Cognitive Function

Three studies investigated the effect of walking on cognitive function. One study provided instructed walking to children in the park, downtown, and in the neighborhood on different days and assessed their concentration by using Digit Span Backwards scores. It was confirmed that walking in the park significantly improved attention and working memory, rather than walking downtown or in the neighborhood [65]. Another study found that groups walking in bamboo forests showed significantly higher attention mean scores than those walking in cities [62]. In another study, the same participants walked across a forest and an urban roadside for 50 min on different dates, and the Visual Backward Digit Span Test (vBDS) was performed. There were no significant differences in vBDS scores between urban and forest walks [53].

One study investigated the effects of exercise on cognitive restoration [55]. Calogiuri et al. [55] performed the same exercise indoors and at parks for 45 min for three days and found significant improvements in the PRS scores in the park exercise group.

Four studies examined the effect of indirect exposure on cognitive function [59,60,61,64]. McAllister et al. [59] found that watching the wild forest and urban forest had significantly higher PRS scores for the forest group than the control group, indicating that viewing images of forests can also mitigate cognitive fatigue. Golding et al. [61] demonstrated that watching a 11 min slideshow of still images of the forest increased the PRS scores significantly compared with a slideshow of the city’s roads or groups that saw nothing. Ng et al. [64] measured the score of the Montreal Cognitive Assessment (MoCA)—an indicator of cognitive function—before and after horticulture sessions for three months and found no significant improvements. Valtchanov et al. [60] compared the mental arithmetic score in groups that observed forests or abstract painting through VR for 10 min and reported that there was no significant difference in the scores between the two groups.

### 3.3. Physiological Outcomes

#### 3.3.1. Nervous System

Two studies investigated the physical response after staying in the forest using indicators of relaxation such as heart rate variability, pulse rate, and cerebral activity [44,66]. Tsunetsugu et al. [44] conducted a 15 min view of a landscape sitting in a city or forest. They found that viewing forests significantly lowered pulse rates. Furthermore, a relaxation effect in the autonomic nervous system was evident. The ln HF value, which is the natural logarithm of the high-frequency power used as an index of parasympathetic nervous system activity, showed an overall high and increasing tendency. The ln LF/(LF+ HF) values, the ratio of low-frequency power used as an indicator of sympathetic nervous activity, showed an overall low tendency. Park et al. [66] also reported results of 20 min of sitting in a city or forest and looking at a landscape. They found a tendency to increase cerebral activity (t-Hb concentration) in the city while decreasing cerebral activity (t-Hb concentration) in forest environments, indicating that staying in the forest is suitable for rest.

Nine studies investigated the effects of walking on physiological relaxation [19,44,49,50,57,62,66,67,68]. Song et al. [49] reported that the average values of heart rate and ln LF/HF when walking in the forest were significantly lower than when walking in the city, whereas the average value of ln HF was significantly higher. Song et al. [50] found that ln HF significantly increased only when walking in the forest, and heart rate (HR) significantly decreased. In addition, the ln HF average was significantly higher in the forest, and the HR average of the forest was significantly lower than in the city. Similarly, Tsunetsugu et al. [44] demonstrated the average value of ln LF/(LF + HF) when walking in the forest was lower than that of the city, and the average value of ln HF was higher than that of the city. However, there were no noticeable changes in HR. Brown et al. [67] compared the results of the forest walking group, the built environment walking group, and the waiting list control group before and after intervention by walking for 20 min twice a week for 8 weeks. They found no significant differences in ln HF and HR. Mao et al. [19] assigned subjects to walk in a city or forest for 7 days, 3 h a day, and found no significant change in HR values before and after walking in both the environments. Zeng et al. [68] conducted interventions of walking for 15 min per day for three days in a city or bamboo grove. They confirmed relaxation effect by observing significantly lower HR levels and significantly higher peripheral oxygen saturation (SpO2) in the group that walked through the bamboo grove. Olafsdottir et al. [57] conducted forest walking, gym walking, and watching a video of a walk in the forest for 40 min and examined the acute and chronic stress responses of each group. They observed an increase in HR and a decrease in ln HF values as a result of walking in nature, which is thought to be due to physical efforts required to walk in nature. Park et al. [66] conducted urban or forest walking interventions for 20 min and found that walking in the forest significantly mitigated against cerebral activity (t-Hb coordination). Hassan et al. [62] repeatedly measured the high alpha wave of groups walking in bamboo groves and urban environments for 15 min. They observed the high alpha wave was significantly higher in the group walking in the bamboo grove than the city, indicating that walking in the forest has a positive effect on brain activity and relaxation.

One study investigated the effect of exercise on physical relaxation [69]. Niedermeier et al. [69] compared before and after exercising in the forest, indoor treadmill walking, and sedentary control for 3 h each and reported that exercise in forests did not cause significant changes in HR values before and after the intervention compared with sedentary controls.

One study investigated the effects of indirect experience on physiological relaxation [60]. Valtchanov et al. [60] investigated the relaxation effect of observing forests or abstraction through VR for 10 min after a stress-inducing task. They found that skin conductivity response was significantly lowered when the forest was observed; however, there was no significant change in HR.

#### 3.3.2. Stress Hormone

Three studies examined the effect of staying on stress hormone reduction [44,56,66]. Tsunetsugu et al. [44] conducted a 15 min view of a landscape sitting in a city or forest, and reported that salivary cortisol level was significantly lowered when looking at the landscape from the forest. Similarly, Park et al. [66] conducted a 20 min sedentary watching in a city or forest. They found that salivary cortisol increased in urban environments, whereas salivary cortisol levels were maintained in forest environments. Rodiek [56] showed observations at the horticultural garden further significantly reduced salivary cortisol levels compared with indoor condition.

Five studies examined the effects of walking on stress hormone reduction [24,48,57,58,66]. Olafsdottir et al. [57] conducted forest walking, gym walking, and watching forest walking for 40 min each in three groups and examined the acute and chronic stress responses of each group. They found that, under chronic stress conditions such as the test period, walking in nature significantly lowered salivary cortisol levels. Jia et al. [24] conducted 1.5 h walk for three days in forests and cities, and demonstrated walking in forests significantly lowered cortisol and epinephrine levels rather than walking in cities. Mao et al. [48] conducted walking for 3 h in cities or forests for three days and showed the serum cortisol levels decreased significantly in the forest walking group, whereas no significant decrease in testosterone levels was found. Grazuleviciene et al. [58] conducted an intervention that took 30 min to walk through an urban park or a city street for seven days. They observed a significant decrease in salivary cortisol levels during the early stages of walking in urban parks, whereas walking on urban streets showed no significant effect. Conversely, Park et al. [66] observed no significant reduction in salivary cortisol during walking in the forest compared with the urban walk.

Two studies examined the effect of exercise on stress hormone reduction [55,69]. Niedermeier et al. [69] reported that exercise in the forest significantly reduced salivary cortisol compared with the sedentary control group, but there was no significant difference compared with indoor exercise. Calogiuri et al. [55] compared the effects of executing the same exercise in a park or indoors for three days for 45 min per day. They found no significant difference in serum cortisol when exercising in the park, but positive improvements were observed in the cortisol wakening response.

One study investigated the effects of indirect exposure to the forest on stress hormone reduction [64]. Ng et al. [64] reported that no significant difference in serum cortisol and dehydroepiandrosterone after three months of sessions in indoor facilities and outdoor forest gardens compared with the waitlist control group.

#### 3.3.3. Blood Pressure

One study investigated the blood pressure lowering effect of staying in the forest [44]. Tsunetsugu et al. [44] demonstrated that sendentary landscape veiwing in a forest led to significantly lower diastolic blood pressure (DBP) than spending time in the city.

Eight studies examined the blood pressure lowering effect of walking in the forest [19,44,49,58,62,67,68]. Mao et al. [19] conducted 3h walking on a forest road at an unhurried pace for seven days, and found a significant decrease in SBP and DBP compared with walking in a city. Brown et al. [67] observed that a group that walked along the path with trees and greenery for 20 min, twice a week for eight weeks, showed a significant decrease in DBP, but no significant decrease in SBP was found. Zeng et al. [68] observed significantly lower SBP in groups that performed 30 min of bamboo grove walking over three days than in groups that performed urban walking, but DBP did not show significant differences. Grazuleviciene et al. [58] conducted a 30 min supervised walk in an urban park or on an urban street for seven days. A significant reduction in DBP in the urban park walking group compared with walking in an urban street was found; however, a significant decrease in SBP was not observed. Hassan et al. [62] performed a 15 min walk in a bamboo grove or city. Both SBP and DBP significantly decreased when walking in the city, whereas DBP and SBP increased while walking in the bamboo grove. Song et al. [49] reported that walking in artificial forests for 15 min did not cause significant changes in blood pressure compared with walking in cities. Similarly, Tsunetsugu et al. [44] reported that walking in a forest for 15 min did not cause significant changes compared with walking in a city.

Three studies investigated the blood pressure lowering effect of exercise in the forest [54,55,69]. Calogiuri et al. [55] reported that groups that performed green exercise in parks showed a significant decrease in DBP compared with indoor exercise groups, but showed no significant change in SBP [55]. Muller-Riemenschneider et al. [54] reported that exercise in urban parks had no significant effect on SBP and DBP compared with standard indoor exercise. Niedermeier et al. [69] reported that exercise in forests caused an increase in DBP and SBP compared with indoor exercise or sedentary groups.

#### 3.3.4. Inflammation

One study investigated the effect of staying on the mitigation of inflammation [45]. Im et al. [45] reported that 2 h of staying in urban and forest areas resulted in a significant decrease in interleukin-8 (IL-2) and tumor necrosis factor α (TNF-α) levels compared with city staying; no significant change in interleukin-6 (IL-6) was reported.

Five studies investigated the effects of walking on the relief of inflammation [17,18,19,24,48]. All five studies compared the results of forest walking and urban walking and confirmed positive effects for forest walking; however, different indicators showed significant changes in each study. Mao et al. [48] reported a significant decrease in IL-6 and TNF-α levels after walking in forests. Mao et al. [18] observed a significant decrease in TNF-α levels after forest walking, but did not observe significant changes in IL-6 levels. Three other studies observed significant reductions in IL-6 levels, but did not observe significant results for TNF-α [17,19,24]. Among the three studies, Jia et al. [24] also reported significant reductions in interferon gamma, interleukin-8, interleukin-1β, and C-reactive protein levels through forest walking, whereas Mao et al. [17] reported no significant reduction in high sensitive-reactive protein levels during forest walking.

#### 3.3.5. Oxidative Stress and Antioxidant

One study investigated the effect of staying on enhancing antioxidant function [45]. Im et al. [45] demonstrated that 2 h of staying in a forest significantly improved antioxidant power (GPx) compared with the urban environment.

Four studies investigated the effect of walking on relieving oxidative stress and improving antioxidant function [17,18,48,63]. Three studies showed that walking in forests reduces malondialdehyde (MDA), an indicator of oxidative stress, more significantly than walking in cities [17,18,48]. Three studies also measured the total superoxide dismutase (T-SOD) activity, an indicator of antioxidant power. One study confirmed a significant increase in T-SOD activity [17], whereas the other two studies did not [18,48]. Chun et al. [63] observed a significant increase in BAP, an indicator of antioxidant power, in the forest walking group rather than in the urban walking group.

#### 3.3.6. Immune Function

One study investigated the effect of staying on promoting immune function. This study assessed the effect of sitting and viewing a landscape for 15 min in a forest, and the results showed that sitting in the forest did not cause significant changes in Ig(A) [44].

Three studies investigated the effects of walking on immune promotion [24,44,48]. Jia et al. [24] conducted three-hour daily walking interventions in cities and forests for patients with chronic obstructive pulmonary disease (COPS). There were significant reductions in perforin levels (NK cells, NK-like cells, and CD8+ T-cells) in the forest walking group, confirming the improved health status of patients with COPS. Mao et al. [48] conducted three-hour daily walking interventions in a city or forest for three days for healthy college men. A significant increase in total B cells in the population performing forest walking was found, but no significant effect was found on the indicators of total T cells, lymphocytes, lymphocytes, NK cells, and CD4/CD8. Tsunetsugu et al. [44] reported that walking in a forest for 15 min had no significant effect on Ig(A) compared with walking in a city.

### 3.4. Methodological Quality Assessment

As a result of the risk of bias assessment of the 32 included studies using RoB 2, 3 studies were evaluated as being of some concern, and 29 studies were at high risk (Table 5).

Regarding the randomized process (D1), most studies reported baseline differences, proving that no problems were caused by randomization. In total, 10 studies reported a detailed randomization process, whereas 22 studies did not explain the process. In addition, 8 studies reported allocation concealment in detail, whereas 24 studies did not report the processes. Thus, 24 studies were evaluated as having some concerns or high risk in the randomized process.

Regarding dropout during an intervention (D2), participants and research attendants in most studies were aware of the assigned intervention. Only two of the 32 included studies were double-blind. Ten studies were single-blind, including three studies with participant being blinded and seven studies with research attendants being blinded. Furthermore, 20 studies did not use blinding. In addition, 14 studies reported that dropout occurred in the middle of the intervention. In total, 7 studies proved that dropout was irrelevant to the trial context or took appropriate analyses to estimate the effect of assignments, whereas 7 studies did not. Therefore, 22 studies were evaluated as having some concerns.

Regarding missing outcomes (D3), 18 studies reported data from all or almost all participants and were evaluated as low risk. A total of 7 studies were evaluated as low risk by providing evidence, performing corrections, or proving that no bias occurred due to the missing outcome. Overall, 7 studies without explanation of potential bias were evaluated as having some concerns as there was uncertainty as to whether the missing value was affected by its true value.

Regarding outcome measurement (D4), 31 studies pre-specified measurements or provided evidence for high validity or high sensitivity of the measurement, whereas 2 studies did not provide sufficient evidence supporting their measurement. In all studies, there was no difference between the intervention and control groups. Overall, 5 studies that included blinding were evaluated as low risk as assessors did not know about the intervention. In total, 5 studies without blinding were evaluated as having some concerns as, even though the assessor knew of the intervention, it was unlikely to affect the result. A total of 22 studies were evaluated as high risk, including self-reported measurements without participant blinding.

Regarding selecting results (D5), one of the 32 studies was evaluated as low risk as it provided a protocol that confirmed the result analysis method before unblinding the outcome data. In 11 studies, all results were reported but were evaluated as having some concerns as they did not provide a sufficient basis for selecting an analysis method. Overall, 20 studies that reported part of the results were evaluated as high risk.

**Table 3:** Main characteristics of included studies.

First Author and Year	Participants	N	Female (%)	Mean Age	Activities	Undertaken Area	Duration	Comparison Group	Outcome Measurement	Study Design
Intervention undertaken in the urban forest										
Ameli 2021 [52]	Participants form military facility	12	25%	35.00	Instructed walking	Woodland road	20 min	Urban road	Distress: DT (+); Mindfulness awareness: MAAS (+)	Randomized cross over
Brown 2014 [67]	Healthy office workers (18–65)	73	21%	42.00	Walking	Nature walking route (trees, grassland, public footpath, country lane)	Twice 20 min/week (for 8 weeks)	(n = 27) Built walking route group (n = 19) W Table 3 and Table 4 are summaries of the overall results, and since we wanted to present the health effects first and then show the tables, it was unavoidable to place the tables away from where we mentioned the hazard tables. aitlist control group	Mental health: SF-8(general health (/), physical health (/), mental health (+)) HRV: ln HF (/); Heart rate (/); Blood pressure: Systolic (−), Diastolic (+); Cardiovascular disease risk biomarker: Framingham CVD risk score (/);	RCT
Calogiuri 2015 [55]	Office workers	14	50%	49.00	Green exercise session (biking bout and a circuit-strength sequence)	Park close to workplace	45 min (3-day workplace session)	Indoor	Affective state: PAAS (PA (+), NA (/) Tranquility (/), fatigue (/)); Restorativeness: PRS (Being away (+); Fascination (+)); Blood pressure: SBP (/), DBP (+); Cortisol awakening response: CAR AUC_G_ (/), CAR AUC_I_ (+); Serum cortisol (/)	RCT
Faber 2009 [65]	Children with ADHD	17	88%	9.23	Carefully controlled, individual, guided walks	Park	20 min	(n = 7) Downtown (n = 4) Neighborhood	Attention and working memory: DSB (+)	RCT (single-blind controlled trials)
Grazuleviciene 2016 [58]	Coronary artery disease patients (45–75)	20	35%	62.30	Supervised 30 min walking	City park (70% of land covered with pine)	30 min (7 days)	(n = 10) Urban street	Mood states: PANAS (PA (/), NA (+/); Blood pressure: SBP (/), DBP (+); Salivary cortisol (+/)	RCT
Muller-Riemenschneid 2020 [54]	Healthy middle-aged adults	145	79%	51.10	Face-to- face park prescription and invitation to weekly green exercise session	Urban park	150 min/week (for 6 months)	(n = 80) Standard physical activity material	General well-being: SF-12(/), WHO-5(/); Distress: K-10(/); Quality of life: WHO QoL (+/); Cardio-metabolic health: blood glucose (/), blood liquids (+/); Blood pressure: SBP (/), DBP (/)	RCT
Ng 2018 [64]	Aged Adults (61–77)	59	78%	67.10	Horticultural therapy weekly session	Indoor, garden	Once a week (for 3 months)	(n = 30) Waitlist control	Psychological well-being: Ryff’s scales of psychological well-being (/); Depression: SDS (/); Anxiety: SAS (/); Social connectedness: Positive relations with others (+), Friendship Scale (/); Satisfaction with Life Scale (/); Inflammatory cytokine: IL-6 (+), IL-1β (/), HCRP (/), Sgp-130(/); Stress: cortisol (/), DHEA (/); Cognitive function: MoCA (/)	RCT
Rodiek 2002 [56]	Aged adults (71–98)	16	100%	84.70	Single instructed session (observing surrounding)	Outdoor horticultural garden	2.5 h	(n = 10) Indoor	Mood states: Philadelphia Geriatric Center Positive and Negative Affect Rating Scale (PA (+/) NA (+/)); Anxiety: STAI (+/); Salivary cortisol (+);	RCT
Song 2019 [49]	Healthy female university students	60	100%	21.00	Walking along a given course (1 km)	Secondary forest or artificial forest	15 min	City area	Mood States: POMS (anxiety (+), depression (+), anger (+), fatigue (+), confusion (+), vigor (+)) Anxiety: STAI (+) HRV ((ln HF (+), ln (LF/HF) (+)); Heart Rate (+); Blood Pressure (/)	Randomized cross over
Song 2018 [51]	Male university students of Japan	585	0%	21.70	15 min walking along a given course	Well-maintained forest area (52 sites)	15 min	City area	Mood states: POMS (depression (+), anxiety (+), anger (+) fatigue (+), confusion (+), vigor (+))	Randomized cross over
Stigsdotter 2018 [47]	Patients with stress-related illness	76	76%	46.40	Nature-based therapy sessions (awareness exercise, nature-based activities, reflection, relaxation)	Forest garden (1.4 ha)	3 h × 3 day/week (for 10 weeks)	(n=) Cognitive Behavioral Therapy	Psychological well-being: PGWBI (+); Burnout: SMBQ (+)	RCT
Intervention undertaken in the forest										
Chun 2017 [63]	Patients with chronic stroke (36–79)	59	32%	60.80	Staying at a recreational forest site (meditation, experiencing the forest through five senses, walking)	Forest	4-day program	(n = 29) Urban hotel	Depression: BDI (+), HAM-D17(+); Anxiety: STAI (+); Oxidative stress: d-ROMs (/); Antioxidant: BAP (+)	RCT
Hassan 2018 [62]	Healthy university students (19–24)	60	50%	19.60	Walking along a given track	Bamboo forest	15 min	(n = 30) City area	Anxiety: STAI (+); Attention: Meditation and attention mean scores (+); Blood pressure: SBP (+), DBP (+); EEG (+)	Randomized cross over
Im 2016 [45]	Young adults (18–35)	41	65%	22.76	Exposure to forest environment	Pine tree forest	2 h	Urban environment	Stress response: SRI-MF (somatic symptoms (+) depressive symptoms (+) anger symptom (/) Total (+)); Inflammatory cytokine: IL-6 (/), IL-8 (+), TNF-α (+); Antioxidant: GPx (+)	Randomized cross over
Jia 2016 [24]	Elderly patients with chronic obstructive pulmonary disease (COPD)	18	33%	70.06	Forest bathing trip (short, leisurely walk in forest)	Forest	1.5 h × 2/day (for 3 days)	(n = 8) Urban	Mood states: POMS (anxiety (+), depression (+), anger (+), vigor (/), fatigue (/), confusion (/)); Inflammatory cytokine: IFN-γ (+), IL-6 (+), IL-8 (+), IL-1β (+), TNF-α (/), CRP (+); Lymphocytes and subsets: NK cell (+), NK-like cell (+), CD8+ T-cell (+); Stress hormones: Cortisol (+), epinephrine (+)	RCT
Koselka 2019 [53]	University students	38	52%	22.90	Walking along forest	Forest preserve	50 min	Urban roadside	Affective states: PANAS (PA (/); NA (+)); Anxiety: STAI (+); Stress: PSS-10(+); Attention and Working memory: vBDS (/)	Randomized cross over
Lee 2014 [15]	Women aged from 60 to 80	62	100%	70.50	Forest walking	Forest	1 h	(n = 19) City	Blood pressure: SBP (+), DBP (+); Cardiovascular disease risk biomarker (arterial stiffness): CAVI (+); Pulmonary function: FEV1 (+), FEV6 (+);	RCT
Mao 2018 [18]	Elderly patients with chronic heart failure who participated in a forest trip 4 weeks ago	20	56%	72.20	Forest bathing trip (3 h walking/day)	Broad-leaved evergreen forest	3 h/day (4-day trip)	(n = 10) City	Cardiovascular disease risk biomarkers: BNP (+); Inflammatory cytokine: IL-6 (/), TNF-α (+); Oxidative stress: MDA (+), TSOD (/)	RCT
Mao 2017 [17]	Elderly patients with chronic heart failure	33	42%	72.20	Forest trip (3 h walking/day)	Forest (Huangyan forest)	4-day trip	(N = 10) City	Mood states: POMS (anxiety (+), depression (+), anger (+), confusion (+), vigor (/), fatigue (/)); Cardiovascular disease risk biomarkers: BNP (+), ET-1 (+), AGT (/), ANG II (/), AT1 (/), AT2 (+); Inflammatory cytokine: IL-6 (+), TNF-α (/), HCRP (/); Oxidative stress: Serum MDA (+), T-SOD (+)	RCT
Mao 2012a [19]	Elderly patients with essential hypertension BP (60 to 75)	24	NA	67.23	Walking at an unhurried pace for 1.5 h × 2/day	Broad leave evergreen forest	3 h/day (for 7 days)	(n = 12) urban area	Mood states: POMS (anxiety (/), depression (+), anger (+), vigor (/), fatigue (+), confusion (+)); Blood pressure: SBP (+), DBP (+); Heart rate (/) Inflammatory cytokines: IL-6 (+), TNF-α (/); Cardiovascular disease risk biomarkers: ET-1 (+), AGT (+), Hcy (+), AT1(+), AT2 (+), Renin (/), ANG II(/)	RCT
Mao 2012b [48]	Healthy male university students	20	0%	20.8	3-day trip including short term forest walking (two 1.5 h walks)	Chamaecyparis obtuse forest	3 h/day (3 days)	(n = 10) Urban	Mood States: POMS (anxiety (+), depression (+), anger (+), confusion (/), vigor (+), fatigue (+)); Inflammatory cytokine: IL-6 (+), TNF-α (+); Oxidative stress: T-SOD (/), MDA (+); Cardiovascular disease risk biomarkers: ET-1 (+), Platelet activation (/); Immunocytes: Total T cell (/), Total B cell (+), Thlymphocyte (/), Tslymphocyte (/), NK cell (/), CD4/CD8(/); Serum cortisol (+), testosterone (/)	RCT
Niedermeier 2017 [69]	Healthy adults (18–70)	42	48%	32.00	Green exercise (mountain hiking)	Forest (Innsbruck region)	3 h	Indoor treadmill walking Sedentary control	Blood pressure: SBP (−), DBP (−); HRV (/); Salivary cortisol (+/)	Randomized cross over
Olafsdottir 2020 [57]	Healthy university students	67	69%	24.39	Forest walk	Recreational forest area of Reykjavík city	40 min	(n = 30) watching forest-walk video (n = 30) Trade mill walk	Affective states: PANAS (PA (+), NA (+)); Salivary cortisol (+); Heart rate (−/); HRV (−/)	RCT
Park 2007 [66]	Male university students	12	0%	22.80	Forest bathing (20 min walk around the given area, 20 min sit and watching the landscape)	Forest area (Seiwa Prefectural Forest)	40 min	City area	Cerebral activity(relaxation): t-Hb concentration (+); Salivary cortisol (+/);	Randomized cross over
Shin 2012 [70]	Alcoholics	92	9%	45.25	Forest therapy camp in recreational forest (Nature–game, nature–interpretation, Mountain-climbing, tracking, orienteering, Nature-meditation, Counseling in forest environment)	Forest (Saneum Recreational Forest)	9-day forest healing camp	(n = 45) Normal daily routines	Depression: BDI (+)	RCT
Song 2015 [50]	Middle-aged hypertensive males	19	0%	58.00	Instructed walk along a given course	Forest environment	17 min	Urban environment	Mood States: POMS (anxiety (+), depression (+), anger (+), vigor (+), fatigue (+), confusion (+); Heart rate (+); HRV: ln HF (+)	Randomized cross over
Sonntag-Öström 2015 [46]	Patients with exhaustion disorder (24–60)	78	86%	44.60	Spend the time in solitude in peace and quiet	Boreal forests	Twice 4 h/week (11 weeks)	(n = 43) waiting list control group	Burnout: SMBQ (/); stress: PRQ (/); Fatigue: CIS (/); Self-esteem: SCQ (/); Anxiety and depression: HAD-S (anxiety (/), depression (/))	RCT
Tsunetsugu 2007 [44]	Male university students	12	0%	22.00	Walking and chair watching	Forest (60 min by car)	15 min	Urban	Subjective feeling: Comfortable (+), calm (+), refreshed (+); HRV: HF (+/), LF/(LF+HF) (+/); Blood Pressure: SBP (+/), DBP (+/); Pulse rate (+/); Salivary cortisol (+); IgA(S) (/)	Randomized cross over
Zeng 2020 [68]	University students (19–24)	120	50%	21.46	Viewing landscape (15 min) Walking (15 min)	(N = 60) Bamboo forest (N = 30) Bamboo forest park	30 min (3 days)	(n = 30) Urban environment	Blood pressure: SBP (+), DBP (+/); Heart rate (+); Oxygen saturation: SpO2(+);	RCT
Intervention undertaken indoors (indirect exposure)										
Golding 2018 [61]	Adults	58	78%	21 to 73	Watching slideshows of still images and reflection	Woodland and heathland in Southern England	11 min	(n = 20) Urban street (n = 20) Control	Affective states: PANAS (PA (−/); NA (/)); Restorativeness: PRS (being away (+); fascination (+)	RCT
McAllister 2017 [59]	Adults of Australia (18–75)	220	72%	49.07	Watching a video film	(N = 72) Wild Forest (N = 76) Urban park	2.5 min	(n = 72) Control	Affective states: PANAS (PA (+ only forest), NA (+ both)); Restorativeness: PRS (+ both)	RCT
Valtchanov 2010 [60]	Undergraduate students	22	54%	17–26	Observing forest via virtual reality	Forest	10 min	(n = 10) Observing abstract paintings via VR	Affective states: ZIPERS (PA (+), NA (/)); Stress: SCR (+), heart rate (/); Cognitive function: Mental-arithmetic score (/)	RCT

AGT angiotensinogen, ANG II angiotensin II, AT1 angiotensin II type 1 receptor, AT2 angiotensin II type 2 receptor, BDI Beck Depression Inventory, BDNF Brain-derived neurotrophic factor, BNP brain natriuretic peptide, CAR cortisol awakening response, CAVI cardio-ankle vascular index, CIS Checklist Individual Strength questionnaire, CRP C-reactive protein, DHEA dehydroepiandrosterone, DBP diastolic blood pressure, DSB Digit Span Backwards, DT distress thermometer, ET-1 endothelin-1, GPx glutathione peroxidase, HAD-S Hospital Anxiety and Depression Scale, HAM-D17 Hamilton Depression Rating Scale, HCRP high sensitive-reactive protein, Hcy homocysteine, HRV heart rate variability, IFN-γ interferon gamma, IL-1β interleukin-1β, IL-6 interleukin-6, IL-8 interleukin-8, K-10 Kessler Psychological Distress Scale, MAAS Mindful Attention Awareness Scale, MDA malondialdehyde, MoCA Montreal Cognitive Assessment, NA negative affect, NK cell (CD56+/CD3−), NK-like cell (CD56+/CD3−), CD8+ T-cell (CD3+/CD8+), PA positive affect, PAAS Physical Activity Affective Scale, PANAS Positive and Negative Affect Schedule, PGWBI Psychological General Well-being Index, POMS Profile and Mood State Questionnaire, PRQ Perceived Stress Questionnaire, PRS Perceived Restorativeness Scale, PSS-10 Cohen’s Perceived Stress Scale, RAS Renin-angiotensin system SAS Self-Rating Anxiety Scale, SCR skin conductance response, SBP systolic blood pressure, SCQ Self-Concept Questionnaire, SDS Self-Rating Depression Scale, SMBQ Shirom-Melamed Burnout Questionnaire, SpO2 peripheral oxygen saturation, SRI-MF Stress Response Inventory-Modified Form, STAI Spielberger State-Trait Anxiety Inventory, TMD total mood disturbance, TNF-α tumor necrosis factor α, T-SOD total superoxide dismutase, T-SOD total superoxide dismutase, vBDS Visual Backward Digit Span Test, vBDS Visual Backward Digit Span Test, WHO-5 Five well-being Index, WHOQoL WHO Quality of life.

**Table 4:** Psychological and physiological outcomes according to the activities conducted in the included studies.

	Direct Exposure																		Indirect Exposure					
	Staying						Walking						Exercise						Nature/Audiovisual Material					
	+	+/	/	-	%p	%p + m	+	+/	/	-	%p	%p + m	+	+/	/	-	%p	%p + m	+	+/	/	-	%p	%p + m
**Psychological outcome**	**5**	**3**	**6**	**-**	**35.7**	**57.1**	**16**	**8**	**-**	**-**	**66.7**	**100.0**	**1**	**2**	**5**	**-**	**12.5**	**25.0**	**3**	**2**	**7**	**1**	**23.1**	**38.5**
Mood	3	1	3	-	42.8	57.1	7	4	-	-	63.6	100.0	-	-	3	-	0.0	0.0	*-*	*-*	*-*	*-*	0.0	0.0
Affect	-	1	-	-	0.0	100.0	1	2	-	-	33.3	100.0	-	1	-	-	0.0	100.0	1	1	-	1	33.3	66.7
Anxiety	-	1	1	-	0.0	50.0	4	-	-	-	100.0	100.0	-	-	-	-	0.0	0.0	-	-	1	-	0.0	0.0
Depression	-	-	1	-	0.0	0.0	2	-	-	-	100.0	100.0	-	-	-	-	0.0	0.0	-	-	1	-	0.0	0.0
Cognitive function	-	-	-	-	-	-	2	1	-	-	66.7	100.0	1	-	-	-	100.0	0.0	2	-	2	-	50.0	50.0
Well-being/quality of life	1	-	1	-	50.0	50.0	-	1	-	-	0.0	100.0	-	1	2	-	0.0	33.3	-	1	3	-	0.0	25.0
**Physiological outcome**	**6**	**2**	**4**	**-**	**50.0**	**66.7**	**42**	**7**	**22**	**1**	**58.3**	**68.1**	**0**	**4**	**3**	**1**	**0**	**50.0**	**1**	**0**	**3**	**0**	**25.0**	**25.0**
Nervous system	1	-	2	-	33.3	33.3	8	1	4	1	57.1	64.3	-	1	1	-	0.0	50.0	1	-	1	-	50.0	50.0
Stress hormone	2	1	-	-	66.7	100.0	5	2	1		62.5	87.5	-	1	1	-	0.0	50.0	-	-	2	-	0.0	0.0
Blood pressure	-	1	-	-	0.0	100.0	3	3	2	-	37.5	75.0	-	1	1	1	0.0	33.3	-	-	-	-	-	-
Cardiovascular disease	-	-	-	-	0.0	0.0	7	-	7	-	50.0	50.0	-	1	-	-	0.0	100.0	-	-	-	-	-	-
Inflammation	2	-	1	-	66.7	66.7	11	-	5	-	68.8	68.8	-	-	-	-	-	-	-	-	-	-	-	-
Oxidative stress/antioxidant	1	-	-	-	100.0	100.0	5		3	-	75.0	75.0	-	-	-	-	-	-	-	-	-	-	-	-
Immune function	-	-	1	-	0.0	0.0	1	1	-	-	50.0	100.0	-	-	-	-	-	-	-	-	-	-	-	-
Pulmonary function	-	-	-	-	-	-	2	-	-	-	100.0	100.0	-	-	-	-	-	-	-	-	-	-	-	-

+: significant effect on positive outcome; +/: including both significant and nonsignificant effect on positive outcome; /: nonsignificant effect; -: negative outcome; %p: ratio of significant effect on positive outcome (count of “+”/total count); %p + m: ratio of positive outcome including both significant and nonsignificant (sum of “+”and “+/”/total count). The numbers listed in the table are calculated and aggregated individual indicators reported in the study.

**Table 5:** Risk of bias of randomized controlled trials and cross-over trials using the RoB 2 tool.

First Author and Year	D1	D2	D3	D4	D5	Overall		
Ameli 2021 [52]	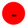	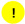	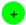	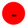	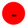	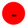	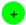	Low risk
Brown 2014 [67]	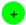	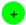	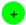	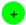	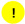	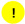	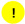	Some concerns
Calogiuri 2015 [55]	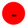	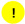	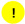	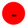	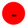	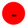	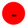	High risk
Chun 2017 [63]	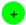	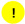	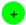	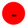	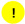	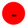	D1: Randomization process	
Faber 2009 [65]	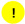	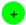	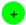	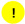	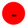	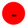	D2: Deviations from the intended interventions	
Golding 2018 [61]	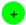	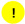	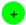	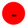	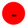	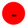	D3: Missing outcome data	
Grazuleviciene 2016 [58]	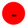	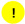	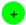	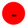	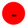	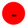	D4: Measurement of the outcome	
Hassan 2018 [62]	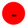	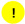	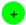	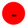	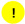	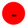	D5: Selection of the reported result	
Im 2016 [45]	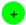	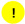	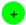	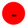	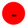	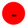		
Jia 2016 [24]	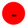	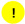	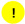	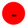	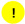	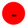		
Koselka 2019 [53]	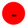	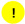	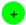	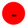	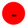	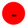		
Lee 2014 [71]	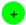	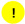	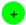	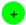	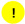	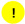		
Mao 2012a [19]	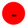	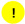	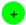	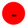	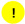	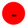		
Mao 2012b [48]	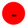	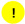	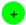	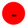	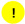	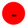		
Mao 2017 [17]	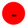	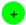	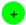	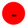	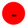	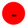		
Mao 2018 [18]	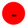	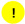	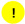	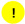	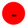	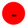		
McAllister 2017 [59]	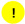	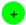	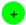	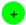	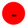	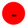		
Müller-Riemenschneider 2020 [54]	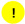	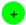	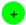	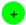	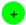	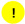		
Ng 2018 [64]	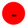	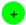	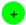	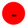	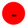	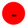		
Niedermeier 2017 [69]	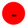	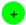	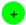	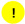	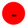	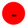		
Olafsdottir 2020 [57]	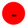	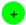	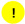	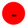	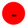	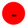		
Park 2007 [66]	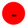	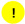	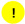	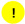	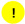	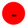		
Rodiek 2002 [56]	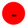	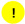	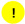	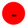	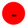	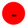		
Shin 2012 [70]	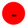	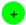	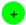	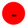	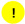	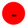		
Song 2015 [50]	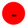	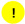	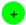	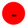	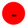	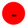		
Song 2018 [51]	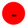	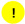	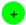	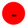	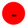	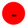		
Song 2019 [49]	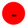	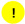	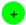	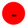	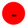	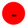		
Sonntag-Öström 2015 [46]	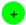	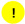	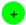	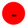	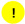	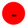		
Stigsdotter 2018 [47]	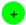	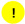	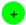	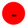	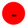	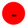		
Tsunetsugu 2007 [44]	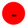	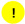	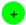	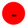	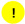	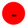		
Valtchanov 2010 [60]	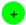	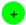	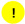	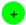	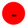	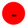		
Zeng 2020 [68]	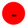	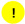	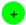	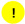	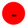	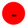		

## 4. Discussion

As the amount of empirical evidence increases, forest-based interventions have been recognized as a new option for preventing disease and improving public health in several countries. Existing reviews have primarily evaluated the overall effectiveness of forest-based interventions themselves. A better nuanced approach is now required to consider the link between each intervention component and health impact. Therefore, this critical review focused on the activity component of forest-based interventions and attempted to identify health impacts according to the activities performed in the program. To this end, recent RCTs accessible from a web database were systematically collected. Although this review does not provide a direct discussion of the long-discussed mechanisms underlying forest-based interventions, it systematically examined the types of activities adopted in recent RCTs and compared the levels of evidence between activities. From this, an up-to-date summary of evidence that could contribute to creating the basis for the design of forest-based interventions was sought to derive.

### 4.1. Activities and Health Effects Investigated in Recent RCTs

The 32 RCTs included in this review were classified according to four activity components: staying, walking, exercise, and indirect exposure. Activities were performed in either an outdoor forest environment or an indoor environment. Outdoor activities were usually performed for a minimum of 15 min, and several authors have noted that a minimum of 15 min are needed to achieve psychological recovery and physiological relaxation [26,44,66]. In contrast, activities using VR materials are mainly conducted within 10 min and were adopted to provide the natural exposure without causing visual fatigue [59,60]. RCTs adopted different activities depending on their focus. Specifically, RCTs for participants with chronic cardiovascular disease adopted low-intensity walking during forest-based interventions [15,17,18,19,24,45,48,58,63,67]. High-intensity exercise has been adopted by healthy participants for the purpose of reducing stress and promoting cardio-metabolic health [54,55,69]. RCTs for psychiatric disorders and stress-related diseases mainly adopted staying during the interventions [46,47]. The number of participants per session was single or about 7 to 12, and depending on the purpose of the intervention, social interaction was limited [46,47,52,56,59] or encouraged [64].

The health outcomes in this review are primarily related to emotional recovery, cognitive restoration, stress reduction, physiological relaxation, and immune function, which reflects an ongoing discussion in the literature that seeks to elucidate the underlying health-promoting pathways of forest-based interventions. Previous studies on forest-based intervention examined cognitive recovery through forest-based interventions based on Attention Restoration Theory (ART; See [72,73]), which explains the mechanisms of directed attention recovery and mental fatigue reduction through interaction with the forest environment [7,74,75,76], or demonstrated arousal of positive emotion and alleviation of negative emotion based on Stress Reduction Theory (SRT or Biophilia hypothesis; See [77,78]), which explains innate preference and aesthetic response towards forest scenery [44,71,74]. Moreover, there has been an increasing number of investigations on the physiological relaxation and immune-strengthening effects of forest therapy [7,14,44,66,79,80], and some studies have examined the benefits of forest chemicals on human health [8,68,81,82,83,84,85,86].

### 4.2. Evidence for the Link between Each Activity and Health Effects

Overall, walking showed the most consistent evidence while staying, exercise, and indirect exposure presented mixed evidence including both significant and nonsignificant outcomes. Regarding psychological outcomes, the consistency of evidence was high in order of walking, staying, indirect exposure, and exercise. In particular, walking showed consistent effects especially on alleviating anxiety, depressive symptoms and negative mood states. Regarding physiological outcomes, the consistency of evidence was high in order of staying, walking, indirect exposure, and exercise. Specifically, walking and staying showed relatively consistent effects on reducing stress hormones and relieving inflammation. Moreover, staying showed more consistent effects on reducing stress hormone levels. This is similar to the results of a meta-analysis that reported the effects of staying (−0.10 μg/dL) and walking (−0.05 μg/dL) in the forests on cortisol level [87].

### 4.3. Identified Knowledge Gaps

There was a significant difference in the number of activities performed. Most studies focused on walking (63%) and staying (19%), and a small number of studies performed exercise and indirect experiences during forest-based interventions. This might be the cause of lowering the consistency of the evidence for both activities. Therefore, future RCTs should be conducted to fill gaps in reported activities. In particular, RCTs on activities with moderate or greater intensity is needed. Moreover, activities can be diversified based on not only the strength of physical activity but also sensory use [88], features of the forest environment [89], and the degree of social interaction [38,90]. In addition, one of the included studies found that indirect nature exposure in comfortable indoor condition is more effective for relieving acute stress than outdoor intervention (opposite in the case of chronic stress) [57]. Activities in nature generally require a higher amount of physical effort than indoors [91,92] and the restorative experience can be hampered in uncomfortable, dangerous, cold, or humid conditions. Therefore, depending on the target effect and the participant, indirect exposure may be more effective. Further studies on the effects of indirect natural exposure (e.g., window view, VR, etc.) are needed.

There were also differences in duration and frequency. Most studies performed short-term interventions within 60 min (63%). Few studies have investigated interventions longer than 1 h, and RCTs that included day-and-nights visits conducted interventions mainly for 180 min. Therefore, RCTs of diverse duration and frequency are needed. Particularly, there are few RCTs on forest-based interventions for 1–3 h or longer and on forest-based interventions performed on a regular basis. Addressing these literature gaps is essential as duration and frequency are crucial features when constructing forest-based interventions.

### 4.4. Improving the Level of Evidence for Forest-Based Interventions

Several challenges were identified in conducting RCTs on forest-based interventions and made suggestions. Above all, most of the included studies were rated at high risk of bias. RoB 2, a reliable tool to evaluate RCT and randomized crossover studies, was used for methodological quality assessment and most of the included studies rated at high risk, especially in the randomization process (D1), outcome measures (D4), and selection of reported outcomes (D5). However, our assessment may not fully reflect the actual quality of the included studies. A number of clinical trials of forest-based interventions are likely to inevitably have a higher risk in the D1 or D4 due to the inherent nature of the intervention rather than the shortcomings of the study design. Forest-based intervention requires exposure to and immersion in the forest; therefore, in a few cases, visiting a therapeutic forest or the instruction of forest therapy specialists is required. Furthermore, days-and-nights programs require accommodation located in the forests. This inevitably makes the allocation of concealments and participant blinding even more difficult, which may result in a higher risk of bias during the randomization process (D1). Additionally, the effects of forest therapy on mood, depression, anxiety, and emotional states are typically measured using self-report methods. This type of measurement without participant blinding may be a major cause of a higher risk of apparent bias in outcome measures (D4). Furthermore, in this review, 11 studies reported the overall results without justifying their analysis method used. This may have led to a higher risk of bias in the reported results (D5). A fundamental way to lower the risk of bias with respect to D5 is to release a protocol that states the analysis method in advance. In addition, it is necessary to establish a standardized monitoring system or institutional guidelines for forest-based interventions to lower the risk of bias and strengthen the evidence for future research.

Alternatively, researchers can focus on the work to improve the quality of the evidence. In addition to the risk of biased evaluation, there are several conditions that provide high-quality evidence [93,94,95]. The quality of evidence can be raised when individual studies reported a large effect or dose–response. A large effect means large effect size or narrow confidence interval. To raise the quality of future RCTs, researchers may try to increase the effect size and reduce the width of the confidence interval by securing a sufficient number of participants, removing potential confounding factors. The dose–response refers presence of dose–response changes. Reporting of differences in the intensity of health outcomes according to exposure can increase the reliability of the observed outcomes and increase the level of evidence. Future RCTs on forest-based interventions may contribute to establishing a strong evidence base by reporting dose–response outcomes, such as effects according to exposure duration, the naturalness of exposure environment, and activity intensity.

Additionally, it was found that most studies conducted the same activity in urban and forest environments. Although the effects of forest-based interventions can be examined by adopting an urban comparator, it may be more suitable to assess environmental effects rather than differences in activities. Therefore, a proper control design is required for future research. For example, a few of the included studies have compared the effects of other activities in the forest [44,66], whereas others have investigated the effects of the environment and activity separately by setting more than one comparator [57,67,69]. Other recent studies have evaluated the therapeutic effect of forest-based interventions by comparing the effect with verified therapy [47,54]. Moreover, staying, walking, and exercising are physical activities that can bring health benefits even if they are not necessarily carried out in the natural environment. Two of the included studies reported no significant differences in performing activities in forest settings compared with indoor settings [57,69]. It is necessary to ascertain whether health outcomes can be considered the effects of forest-based interventions rather than mere effects of physical activity. Furthermore, result measurement should be determined according to the characteristics of activity. When performing simple activities such as walking or staying, participants can focus primarily on their surroundings. However, when engaging in complex activities such as high-intensity exercise or learning, participants have difficulty concentrating on their surroundings. This may bring a risk of compromising the restorative mechanisms from nature exposure. Taking this into account, when the physical or cognitive demands of a program are high, it would be beneficial to examine long-term outcomes (e.g., probability of activity continuing, long-term stress relief, and increased resilience) rather than short-term effects of activities [55].

## 5. Conclusions

The purpose of this review was to identify the types of activities currently being performed in RCTs for forest-based interventions and to examine the association between health benefits and each activity component. This review synthesized 32 studies and categorized activities into staying, walking, exercise, and indirect exposure. Walking showed the most consistent effects, whereas other activities showed mixed effects of both significant and nonsignificant results. In the psychological results, walking showed stable effects on relieving depression and anxiety symptoms. In the physiological results, staying and walking showed relatively consistent effects on reducing stress hormone levels and relieving inflammation.

However, most of the included studies rated at high risk of bias. Therefore, appropriate blinding, protocol registration, and standardized monitoring systems for forest-based interventions should be developed. Additionally, future RCTs need to diversify activities, duration, and frequency of interventions to fill existing literature gaps. In addition, appropriate control design, measurement methods, elimination of potential confounders, and dose–response investigation should be considered to improve the quality of evidence linking activities and health effects.

Today, the health benefits of forests have started to expand from empirical knowledge to preventive medicine in practice. Along with an increasing public demand for recreation and health use of forests, it is necessary to provide reliable and effective forest-based intervention. Although this review had some limitations, it is expected that the findings highlighted here will contribute to future evidence-based designs for forest-based interventions.

## Figures and Tables

**Table 1:** Eligibility criteria for study selection.

PICOS	Inclusion Criteria	Exclusion Criteria
Population	Studies with healthy or unhealthy humans	Studies not including human participants.
Intervention	Studies reporting any intervention that matched our definition of “Designed and structured activities which utilize a defined green space—park, urban forest, and forest—as a health promotion tool”.	Studies not including designed or structured interventions. Studies not providing a description of the green space where the intervention was held.
Comparator	Studies with a comparison group (e.g., waitlist group, urban group, normal daily routines, other comparative intervention).	NA
Outcome	Any quantitative psychological and physiological outcome at an individual level related to health and well-being.	Studies not including health and well-being outcomes. Studies not including quantitative outcomes. Studies including only population-level or community-level outcomes.
Study Design	Randomized controlled trials and randomized cross-over studies.	Reviews, qualitative studies, nonrandomized controlled trials, uncontrolled before and after, with no comparator groups relevant for the current review, and quasi experiments.

**Table 2:** Search keywords.

PICOS	Keywords
P	(“people” OR “volunteers” OR “participants” OR “subjects” OR “individuals”)
I	(“natural environment” OR “green space” OR “nature space” OR “green nature” OR “forest”)
	AND
	(“intervention” OR “program” OR “programme” OR “exposure” OR “therapy” OR “recreation” OR “physical activity” OR “exercise” OR “activities” OR “walking” OR “meditation” OR “staying”)
C	NA
O	(“health” OR “well being” OR “well-being” OR “health promotion” OR “physiological” OR “psychological” OR “mental health” OR “physical health” OR therapeutic)
S	(“randomized controlled” OR “RCT”)

## Supplementary Materials

The following are available online at https://www.mdpi.com/article/10.3390/ijerph19052692/s1, Table S1: PRISMA 2020 checklist.

## Registration

OSF Registration DOI: 10.17605/OSF.IO/8NYVH.
